# Aspects cliniques et biologiques des anémies pédiatriques dans un hôpital de District urbain au Cameroun

**DOI:** 10.11604/pamj.2013.16.91.3307

**Published:** 2013-11-11

**Authors:** Danièle Kedy Koum, Eveline Ngouadjeu Dongho Tsakeu, Françoise Ngo Sack, Pierre Tchienrg Moueleu Ngalagou, Albert Kamanyi, Samuel Honoré Mandengue

**Affiliations:** 1 Service de Pédiatrie, Hôpital de District de Bonassama. BP: 9023 Douala, Cameroun; 2 Faculté de Médecine et de Sciences Pharmaceutiques, Université de Douala. BP 2701, Douala, Cameroun; 3 Service d'Hématologie, Hôpital Général de Douala, Cameroun; 4 Service d'Hématologie, Hôpital Central de Yaoundé, Cameroun; 5 Unité de Physiologie des Activités Physiques et Sportives, Faculté des Sciences, Université de Douala. BP: 7064 Douala, Cameroun; 6 Laboratoire de Physiologie, Faculté des Sciences, Université de Dschang. BP: 96 Dschang, Cameroun

**Keywords:** Anémie pédiatrique, Prévalence, Etiologie, Douala, Cameroun, pediatric anemia, prevalence, Etiology, Douala, Cameroon

## Abstract

**Introduction:**

L'anémie est un problème de santé publique prédominant chez les enfants et les femmes en âge de procréer. L'objectif de cette étude était de caractériser et de déterminer sa prévalence chez les enfants âgés de 6 mois à 14 ans hospitalisés à l'hôpital de District de Bonassama à Douala-Cameroun.

**Méthodes:**

Il s'agissait d'une étude descriptive transversale qui s'est déroulée de février à mars 2012 avec une collecte rétrospective des données de janvier 2009 à mars 2012. Etaient inclus, les patients de 6 mois à 14 ans hospitalisés, quel que soit leur motif de consultation et leur diagnostic, et ayant réalisé au moins une numération formule sanguine (NFS). Les patients transfusés moins de 120 jours avant la NFS étaient exclus. L’âge, le sexe, les motifs de consultation, les signes cliniques, les diagnostics et les résultats de la NFS étaient enregistrés. L'anémie était définie selon les critères de l'OMS. La recherche étiologique était guidée par les signes cliniques et les examens complémentaires.

**Résultats:**

La prévalence de l'anémie était de 88,5%. Le sex-ratio garçon/fille était de 1,3. La fièvre était le premier motif de consultation. L'anémie modérée prédominait (62,7%). Le taux moyen d'hémoglobine chez les patients anémiques était de 8,6 ± 1,7 g/dl (2,3- 11,4). L'anémie microcytaire hypochrome dominait (48,5%). Le paludisme était la principale pathologie (46,3%). Le taux de mortalité des patients anémiques était de 5,9%.

**Conclusion:**

La prévalence de l'anémie était élevée avec une prédominance des formes hypochromes microcytaires. La principale étiologie était le paludisme.

## Introduction

Selon l'Organisation Mondiale de la Santé (OMS), l'anémie est le problème de santé publique le plus fréquent dans le monde, et touche tous les âges [1–3]. Deux milliards de personnes dans le monde sont concernées et ce sont les pays en voie de développement qui payent le plus lourd tribut avec des prévalences de l'ordre de 60% chez les femmes enceintes, 50% chez les enfants de moins de 4 ans et 45% chez les enfants d’âge scolaire [4, 5]. Au Cameroun, les récentes enquêtes démographiques et de santé ont montré que l′anémie affecte plus de 60% des enfants de moins de 5 ans avec 68% en 2004, et 60% en 2011, résultats classant l′anémie comme un problème de santé publique grave au Cameroun [6–9]. La carence en fer est la cause la plus fréquente d´anémie, mais les carences en micronutriments sont aussi impliquées (acide folique, vitamine B12, vitamine A). Les inflammations qu´elles soient aigues ou chroniques et les parasitoses intestinales sont également des causes fréquentes d´anémie. Les troubles héréditaires ou acquis altérant la synthèse de l´hémoglobine, la production des hématies et leur survie peuvent également entrainer des anémies [9]. L´anémie étant de cause multifactorielle, il est important d´identifier son étiologie pour la prendre en charge efficacement [3, 6].

L'objectif de cette étude était de caractériser et de déterminer la prévalence des d'anémies chez les enfants âgés de 6 mois à 14 ans dans un hôpital de District urbain à Bonassama Douala-Cameroun.

## Méthodes

### Site d´étude et méthodologie

Il s'agissait d´une étude descriptive transversale qui s´est déroulée de février à mars 2012 dans le Service de Pédiatrie de l´Hôpital de District de Bonassama (l´HDB) dans la ville de Douala au Cameroun. Elle a consisté en une collecte rétrospective des données de 515 patients du premier janvier 2009 au 31 mars 2012. Ont été inclus, les patients des 2 sexes, âgés de 6 mois à 14 ans hospitalisé à l´HDB quel que soit leur motif de consultation et leur diagnostic et ayant réalisé au moins une numération formule sanguine (NFS) durant leur hospitalisation. Ont été exclus, les patients remplissant ces critères mais dont la NFS a été précédée d´une transfusion sanguine dans les 120 jours. L´âge, le sexe, les motifs de consultation, les signes cliniques, les pathologies diagnostiquées et les résultats de la NFS ont été enregistrés pour chaque patient sous forme ad hoc. Les NFS ont été effectuées au laboratoire de l´HDB, sur un automate d'hématologie de marque MINDRAY BC-2800 (Shelzhen Mindray Biomedical Electronic CO LTD/CHINA). Les données ont été analysées anonymement. L’étude a été conduite après approbation du comité de recherche de l´hôpital.

### Diagnostic de l´anémie et définitions cliniques des co-morbidités

La définition de l´anémie et de sa sévérité en fonction de l´âge était basée sur les taux limites d'hémoglobine de l'OMS [9]. Le volume globulaire moyen était considéré comme normal entre 80 et 100 fl et la concentration corpusculaire moyenne en hémoglobine était normale entre 32 et 36 g/dl. La recherche de l´étiologie de l´anémie était basée sur des critères cliniques confirmés dans la mesure du possible par des examens complémentaires. Pour le diagnostic du paludisme une goutte épaisse était demandée aux patients présentant de la fièvre. Les gastroentérites étaient affirmées devant des selles liquides ou molles trop fréquentes (en général supérieures ou égales à 3 par jour) associée ou non à des vomissements. L´infection urinaire était confirmée par un examen cytobactériologique des urines (ECBU) effectué lorsque l´enfant présentait un syndrome inflammatoire à réponse systémique (fièvre, tachycardie, tachypnée, hyperleucytose, leucopenie), associé à des troubles digestifs et/ou urinaires. La bronchopneumonie était diagnostiquée chez tout enfant présentant une toux, une polypnée, des râles et/ou un syndrome de condensation. La confirmation par une radiographie du thorax n´était pas systématiquement faite. L´électrophorèse de l´hémoglobine était demandée devant des antécédents familiaux ou des signes cliniques évocateurs de drépanocytose (crises vaso-occlusives). La sérologie VIH était effectuée devant des antécédents familiaux (sérologie HIV positive chez les parents ou la fratrie, décès de parents ou décès dans la fratrie) ou des signes cliniques évoquant une immunodépression (candidose, malnutrition sévère, infections sévères et/ou récidivantes). Elle consistait en un test rapide positif confirmé par un test ELISA (Enzyme Linked Immunosorbent Assay). Pour les enfants de moins de 18 mois, les tests virologiques (PCR: polymerase chain reaction) étaient effectués hors de l´hôpital, et les résultats n´étaient pas disponibles dans les dossiers. L´examen parasitologique direct des selles était effectué devant les troubles digestifs à la recherche des parasitoses intestinales pouvant être cause d´anémie. Le dosage de la CRP était demandé devant un syndrome inflammatoire à réponse systémique. Le dosage de la ferritine n´était pas possible au sein de l´hôpital.

### Analyse statistique

Les données ont été traitées avec le logiciel Microsoft Excel 2007 et analysées à l´aide du logiciel Statview version 5.0 (SAS Institute Inc). Les données quantitatives ont été présentées en moyenne ± déviation standard (SD) et les données catégorielles sous forme d'effectifs et de pourcentages. Entre les différents groupes, les moyennes ont été comparées en utilisant le test-t non apparié et les pourcentages comparés en utilisant les tests de Chi carré et de Fisher. Les différences ont été considérées comme significatives pour p

## Résultats

### Population d´étude et prévalence

Au total, 515 dossiers médicaux de patients âgés de 6 mois à 14 ans ont été étudiés. La prévalence de l´anémie était de 88,5% (456/515). La répartition des patients par tranche d´âge et par sexe est présentée dans le Tableau 1. Les enfants présentant une anémie étaient majoritairement de sexe masculin avec 263 (57,7%) garçons et 193 (42,3%) filles soit un sex-ratio garçons/fille de 1,3. L´âge moyen des patients anémiques était de 2,6 ± 2,7 ans et ne différait pas significativement de celui des enfants non anémiques (2,2 ± 1,8 ans; p=0,23). Globalement, la fréquence de l´anémie n´était pas significativement plus importante pour une tranche d´âge donnée (P=0,09).

**Tableau 1:** Population d’étude

Tranches d’âge	Effectif total des participants	Effectif des enfants présentant une anémie					
		Filles		Garçons		Total	
		N	%	N	%	N	%
6-59 mois	426	158	42,5	214	57,5	372	87,3
5-11 ans	81	32	42,1	44	57,9	76	93,8
12-14 ans	8	3	37,5	5	65,5	8	100
Total	515	193	42,3	263	57,7	456	88,5

N = effectif, % = pourcentage P = 0,09

### Présentation clinique

**Figure 1:**
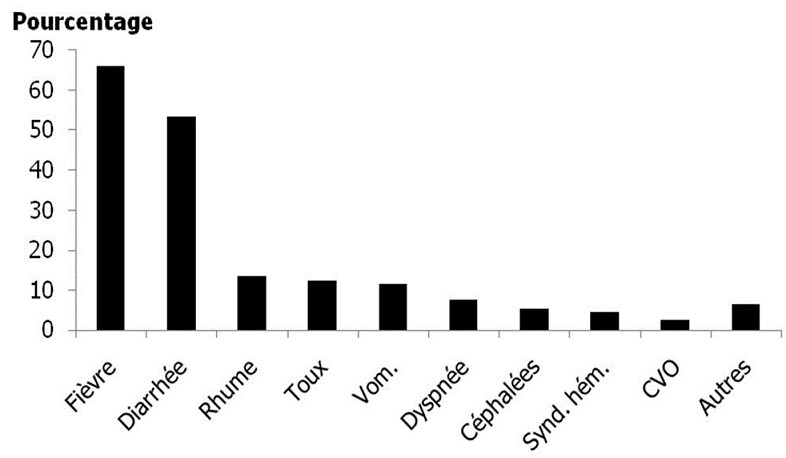
Motifs de consultation

Les principaux motifs de consultation des patients présentant une anémie sont illustrés dans la Figure 1. La fièvre était le premier motif de consultation suivie de la diarrhée. Les principaux signes et symptômes en rapport avec l´anémie étaient la pâleur cutanéo-muqueuse, l´asthénie, la tachycardie, la dyspnée, les céphalées et les vertiges. Page number not for citation purposes 3

### Types d´anémies

Le taux moyen d'hémoglobine chez les patients anémiques était de 8,6 ± 1,7 g/dl (2,3-11,4). Le Tableau 2 montre le degré de sévérité de l´anémie en fonction de l´âge. L´anémie modérée était plus fréquente à toutes les tranches d´âge (62,7%), cependant une différence significative n´a été observée entre les tranches d’âges que pour les anémies légères (p=0,001) et les anémies sévères (p=0,0484).

**Tableau 2:** Degré de sévérité de l'anémie en fonction de l’âge

	Tranche d’âge			
Degré de sévérité	6-59 mois	5-11 ans	12-14 ans	P
	N (%)	N (%)	N (%)	
Anémie Légère	81 (21,8%)	4 (5,3%)	1 (12,5%)	0,0010
Anémie Modérée	230 (61,8%)	51 (67,1%)	5 (62,5%)	0,6867
Anémie Sévère	61 (16,4%)	21 (27,6%)	2 (25,0%)	0,0484

N = effectif, % = pourcentage

Les caractéristiques des différentes anémies sont illustrées dans le Tableau 3 Les anémies microcytaires hypochromes étaient les plus fréquentes (48,5%) suivies des anémies normocytaires normochromes (22,6%). Il n´a pas été observé de différence significative entre les types d´anémie pour les différentes tranches d´âge (p=0,076).

**Tableau 3:** Caractérisation des d'anémies selon le VGM et la CCMH

	Volume globulaire moyen			
CCMH	Microcytaire	Normocytaire	Macrocytaire	Total
Hypochrome	221 (48,5%)	32 (7,0%)	0 (0%)	253 (55,48%)
Normochrome	73 (16,0%)	103 (22,6%)	27 (5,9%)	203 (44,52%)
**Total**	**294 (64,5%)**	**135 (29,6%)**	**27 (5,9%)**	**456 (100%)**

### Diagnostic étiologique

Le paludisme était fréquent chez les patients anémiques (46,3%; p=0,73). Les autres pathologies étaient les broncho-pneumopathies (21,7%; p=0,58), les gastro-entérites (10,5%; p=0,41), les infections urinaires (6,2%; p=0,51) et les parasitoses intestinales (6,5%; p=0,77). Nous n´avons pas observé d´association significative entre ces différentes pathologies et l´anémie. Huit patients ont été diagnostiqués drépanocytaires homozygotes, 17 avaient une sérologie VIH positive. Une protéine C-réactive (CRP) positive a été retrouvée chez 239/396 patients anémiques (soit 60,3%; p=0,019).

### Mortalité

Le taux de mortalité des patients présentant une anémie était de 5,9% et était significativement plus élevé que celui des patients non anémiques (p=0,028).

## Discussion

La prévalence des anémies dans notre étude était de 88,5%. Cette valeur est largement supérieure au seuil de 40% défini par l´OMS pour une endémie sévère d'anémie dans une population [4]. Elle ne reflète cependant pas la prévalence de l´anémie dans la population générale et s'expliquerait par le fait que l’étude a été menée en milieu hospitalier chez des enfants malades pour lesquels les facteurs favorisant l´anémie sont forcément plus fréquents [10].

La fièvre, la diarrhée, le rhume et la toux étaient les principaux motifs de consultation des patients. Ces motifs de consultations étaient en rapport avec les résultats des enquêtes démographiques et de santé de 2004 et 2011 qui montraient qu'au Cameroun, la fièvre, la diarrhée et les infections respiratoires aiguës étaient les problèmes de santé les plus importants chez les enfants [6, 7]. Une étude menée à Yaoundé par Djeutchouang a permis de rapporter une fréquence élevée (74,3%) de la fièvre chez les patients anémiques [11]. Ouédraogo et al. ont retrouvé les mêmes signes au Burkina Faso [12]. Malumba et Muhindo ont retrouvé sur 1645 dossiers médicaux d´enfants de moins de 5 ans hospitalisés dans les hôpitaux de référence de Kinshasa 85,8% des patients avec de la fièvre [13]. La pâleur cutanéo-muqueuse, l'asthénie, la tachycardie et la dyspnée étaient les principaux signes retrouvés chez les patients anémiques. Ces signes ont été observés par Ouédraogo et Khieu respectivement au Burkina Faso chez les enfants de 12 à 23 mois et au Cambodge chez les enfants en âge scolaire [12, 14]. En République Démocratique du Congo, Malumba et Muhindo ont rapporté une prévalence de 38,7% de pâleur cutanéo-muqueuse chez les enfants de moins de 5 ans hospitalisés pour paludisme parmi lesquels 64,9% souffraient d'anémie grave [13]. Ces signes étaient les critères les plus retrouvés du syndrome anémique dans les différentes études.

Le taux moyen d´hémoglobine était de 8,6 ± 1,7 g/dl. Ce taux moyen était proche de 8,2 g/dl trouvés par Ouédraogo et al. au Burkina Faso en 2008 chez les enfants anémiques âgés de 6 à 23 mois [12]. Mbanya et al ont trouvé un taux d´hémoglobine moyen de 7,8 g/dl chez 105 enfants cliniquement anémiques au Centre Hospitalier Universitaire de Yaoundé au Cameroun [15]. El Houi et al. avaient un taux moyen de 10 g/dl chez 85 enfants d’âge préscolaire au Maroc [5]. L´anémie modérée était beaucoup plus fréquente que l´anémie légère et l´anémie sévère, ce profil a été retrouvé dans les différentes enquêtes démographiques et de santé en 2004 et 2011 [6, 7]. L'anémie était en général microcytaire hypochrome suivie par l´anémie normocytaire normochrome. Diagne et al. ont également trouvé une anémie majoritairement de type hypochrome microcytaire à l'issu d'une étude menée en 2010 chez les enfants à Dakar au Sénégal [3, 16]. Au Burkina Faso, 65,1% de patients présentant une anémie et âgés de 6 à 23 mois avaient une anémie hypochrome [12]. Atanda et al. en 1997 ont trouvé par contre une forte prévalence de l'anémie microcytaire normochrome suivie par l'anémie microcytaire hypochrome chez les enfants à Pointe-Noire [17]. Ouédraogo et al. (2008) ont également rapporté que la fréquence des formes hypochromes microcytaires était plus élevée parmi les anémies sévères que parmi les anémies modérées [12].

Plusieurs pathologies ont été retrouvées chez les patients anémiques. Le paludisme, la gastroentérite et les broncho-pneumopathies étaient les pathologies les plus observées. La prévalence du paludisme (46,3%) dans notre échantillon était proche de celle de l´anémie chez l´enfant à l’échelle nationale (40%) lors de l'EDSC-III [6]. Le paludisme a été clairement incriminé dans la survenue de l'anémie chez l'enfant camerounais et africain [18–20]. Djeutchouang a trouvé une fréquence du paludisme de 41,2% [11]. Mbanya et al. ont montré que le paludisme et la drépanocytose jouent un rôle important dans la survenue de l'anémie chez les enfants africains de 5 à 10 ans [15]. Levy et al. ont trouvé au cours d'une étude prospective en 2005 que l'anémie était un facteur indépendant de risque de diarrhée et des maladies respiratoires chez les enfants américains de 7 à 18 mois [21]. Dans notre étude nous n´avons pas observé une association significative entre l´anémie et les différentes comorbidités. Concernant la sérologie VIH et la drépanocytose, le dépistage n´a pas été systématique chez tous les patients, ceci ne nous permettant pas de calculer une prévalence. De plus pour le VIH, nous n´avions pas les résultats des tests virologiques chez les enfants de moins de 18 mois pour confirmer l´infection à VIH chez ces enfants. Le dosage de la CRP a permis d´affirmer la fréquence du syndrome inflammatoire (60,3%) chez les enfants hospitalisés et présentant une anémie, cette inflammation était significativement plus fréquente chez l´enfant anémique que chez l´enfant non anémique. Par contre, bien qu´étant généralement incriminée comme une cause majeure d´anémie chez l´enfant [9], la carence martiale n´a pas pu être objectivée car le dosage de la ferritinémie ne pouvait être réalisé dans le laboratoire de l´hôpital et les parents ayant des ressources limitées n´ont pas pu réaliser cet examen dans les laboratoires externes.

Le taux de mortalité des patients anémiques était de 5,9%, et pouvait s'expliquer par le fait que l’étude a été menée dans un centre hospitalier. Ce taux était significativement plus élevé que celui des enfants du même âge non anémiques (p Page number not for citation purposes 4
